# Supplementary material for: Sustained-input switches for transcription factors and microRNAs are central building blocks of eukaryotic gene circuits
Source: Genome Biol. 2013 Aug 23;14(8):R85. doi: 10.1186/gb-2013-14-8-r85 (PMC4054853; doi:10.1186/gb-2013-14-8-r85)
Supplement: Additional file 5 — HTML Browsable Motif Output. Zipped folder containing all WaRSwap and FANMOD motif output, viewable in a web browser. [file gb-2013-14-8-r85-S5.ZIP › HTML_browsable_motif_output/WaRSwap_ath_tair10/sigs_WSWRSLDraw_TAIR10-2500.pvals.heatmaps.html/motif_id_38_000101101_tftype_ath_upstream_-3000_0.html]

```
BG_MODEL = WARSwap
MOTIF_ID = 38_000101101
TF_TYPE = ath
UPSTREAM = -3000_0


PVals
FNR = 0.2	FNR = 0.4	FNR = 0.6	FNR = 0.8
deltaG = 60	0.127	0.216	0.044	0
deltaG = 70	0.195	0.25	0.022	0.002
deltaG = 80	0.131	0.138	0.007	0.002

ZScores
FNR = 0.2	FNR = 0.4	FNR = 0.6	FNR = 0.8
deltaG = 60	1.211	0.782	1.913	4.969
deltaG = 70	0.938	0.646	2.241	4.809
deltaG = 80	1.231	1.133	2.828	4.768

StDevs
FNR = 0.2	FNR = 0.4	FNR = 0.6	FNR = 0.8
deltaG = 60	62.84	32.803	12.96	4.047
deltaG = 70	58.786	30.791	12.467	4.096
deltaG = 80	53.338	28.729	11.321	4.02
```
